# Risk of fall in patients with chronic kidney disease: results from the China health and retirement longitudinal study (CHARLS)

**DOI:** 10.1186/s12889-024-17982-4

**Published:** 2024-02-16

**Authors:** Pinli Lin, Biyu Wan, Jintao Zhong, Mengya Wang, Fang Tang, Lingzhen Wang, Junjun Guo, Yuling Ye, Xusheng Liu, Lu Peng, Lili Deng

**Affiliations:** 1 The Second Clinical College of Guangzhou University of Chinese Medicine, Guangzhou, China; 2 School of Nursing, Hunan University of Chinese Medicine, Changsha, China; 3 The Second Affiliated Hospital of Guangzhou University of Chinese Medicine (Guangdong Provincial Hospital of Traditional Chinese Medicine), Guangzhou, China

**Keywords:** Falls accident, Chronic kidney disease, CHARLS, PSM

## Abstract

**Background:**

Chronic kidney disease (CKD), often coexisting with various systemic disorders, may increase the risk of falls. Our study aimed to assess the prevalence and risk of falls among patients with CKD in China.

**Methods:**

We included patients with/without CKD from China Health and Retirement Longitudinal Study (CHARLS). Our primary outcome was the occurrence of fall accidents within the past 2 years. To enhance the robustness of our findings, we employed a multivariable logistic regression model, conducted propensity score analysis, and applied an inverse probability-weighting model.

**Results:**

A total of 12,658 participants were included, the prevalence of fall accident rates were 17.1% (2,028/11,837) among participants without CKD and 24.7% (203/821) among those with CKD. In the inverse probability-weighting model, participants with CKD exhibited higher fall accident rates (OR = 1.28, 95% CI: 1.08–1.53, *p* = 0.005 ). Sensitivity and subgroup analysis showed the results still stable.

**Conclusions:**

The population in China afflicted with CKD has a significantly heightened risk of experiencing falls, underscoring the crucial importance of intensifying efforts in assessing and preventing fall risks.

**Supplementary Information:**

The online version contains supplementary material available at 10.1186/s12889-024-17982-4.

## Introduction

The global prevalence of chronic kidney disease (CKD) is exhibiting an upward trajectory, with a incidence estimated at approximately 8.2% in China [1, 2]. The prevalence of CKD demonstrates a linear escalation with advancing age, where the intermediate and elderly age groups constitute more than 60% of the total population affected by this condition [3]. CKD is frequently concomitant with various systemic disorders, often leading to complications such as cognitive impairment, reductions in strength, osteoporosis, muscle wasting, frailty and an increased risk to falls [4–10].

Globally, falls stand as the second most prevalent cause of unintentional injury-related fatalities [11, 12]. Particularly accentuated in China, falls have surged to the forefront as the primary instigator of disability, functional constraints, and even mortality within the middle-aged and elderly demographic. Patients with CKD are often considered at a high risk of falling [13]., due to the frequent occurrence of bone metabolism abnormalities in CKD, these patients are more susceptible to fractures following falls, further impacting their quality of life. Therefore, previous studies have explored and identified the risk factors for falls among CKD patients, including age, History of falls, and multiple comorbid conditions [14–16]. However, there is limited research on the increased risk of falls among CKD patients, and the risk of falls among Chinese chronic kidney disease patients remains uncertain [17]. We therefore conducted this prospective study using the data from China Health and Retirement Longitudinal Study (CHARLS) to assess the risk of fall in patients with chronic kidney disease in China.

## Methods

### Study population and design

The data was extracted from China Health and Retirement Longitudinal Study (CHARLS) [1888888888888888] [18888888888888]. This project is a nationally representative survey of households and individuals aged 45 years and above in China, conducted by the National Institute of Development of Peking University. This survey covers 28 provinces and aims to gather demographic and health-related data on middle-aged and older adults. The CHARLS was approved by the Biomedical Ethics Review Committee of Peking University. All participants provided written informed consent. The ethical approval number of CHARLS is IRB00001052-11015.

**Fig. 1:**
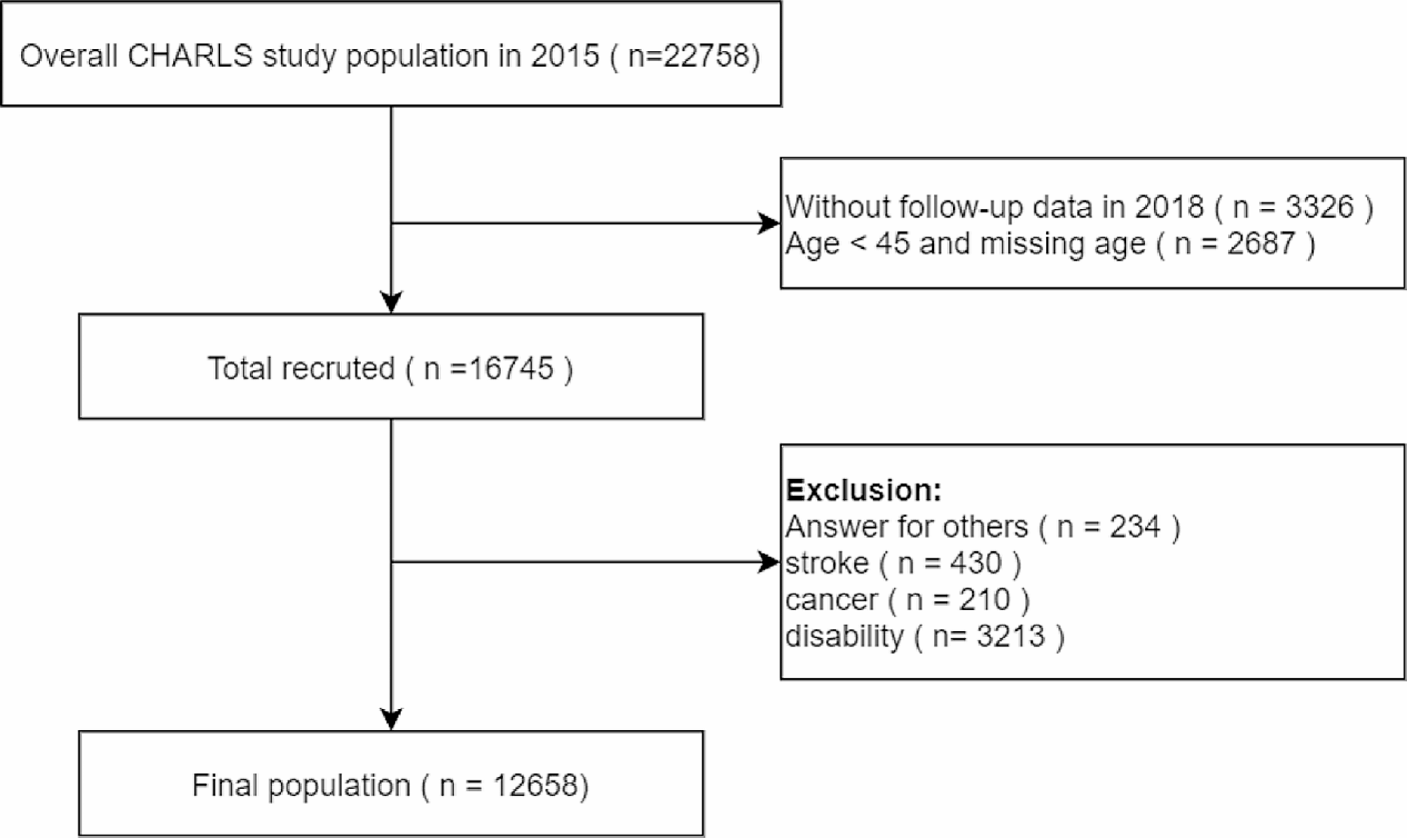
Flowchart of patient selection

The survey in 2015–2016 and the follow up survey in 2017–2018 were included for the analyses in this study. For the current analysis, we restricted the participants to those aged 45 years or above. We also excluded participants without fall down information in 2017–2018 survey. In addition, we excluded participants with missing cognitive data due to answers provided by others, those who had experienced a stroke, cancer, or had disabilities (including physical disabilities, brain damage/mental retardation, or vision problems). This resulted in a final study cohort of 12,658 participants (Fig. 1).

### Chronic kidney disease

The diagnosis of CKD was established through participants’ self-reports of physician-diagnosed conditions at baseline, adhering to professional guidelines. Validation occurred during a subsequent follow-up assessment in 2015, involving direct participant confirmation of the accuracy of the recorded CKD diagnosis from their last interview. This rigorous method aimed to ensure the precision and reliability of the reported data.

### Fall accidents

The primary outcome was fall accidents. Participants self-reported these incidents, which were captured using the following question in the 2017–2018 survey: “Have you experienced any falls since your last visit?” Participants provided binary responses, indicating either “yes” or “no.“,

### Assessment of covariates

Demographic information includes age, gender, alcohol status, pain status, night sleep duration, fall down experience, mobility, activity of daily living (ADL), instrumental activities of daily living (IADL), cognition, depression, and toilet seat usage; while disease history covers self-reported hypertension, dyslipidemia, and diabetes.

The cognitive function assessment in CHARLS includes telephone interviews for cognitive status (TICS-10), a visuospatial ability test, and an episodic memory capacity test. The cumulative total score from these assessments is 21, with a score below 5.25 being indicative of a risk for cognitive impairment [18]. Depression assessment in CHARLS is conducted using the CESD-10 [19]. The cumulative total score is 30, with a score of 10 or higher indicating a high risk of depression. The use of mobility includes walking sticks, travel devices, manual wheelchairs, and electric wheelchairs.

### Statistical analysis

All of the analyses were performed using the statistical software packages R 4.2.2 (http://www.R-project.org, The R Foundation) and Free Statistics software versions 1.7.1. Continuous variables are reported as the mean ± standard deviation, and categorical variables are presented as percentages. Baseline characteristics were compared using t-test for continuous variables and the chi-square test for categorical variables. Missing data was imputed using the random forest method.

To minimize potential treatment allocation bias and confounding, logistic regression with propensity score matching (PSM) was employed to estimate the likelihood of patients experiencing falls. We applied a 1:1 nearest neighbor matching algorithm with a caliper width of 0.01. The propensity score was generated using the following variables: age, gender, alcohol status, pain status, night sleep duration, fall experience, mobility, ADL, IADL, cognition, depression, toilet seat usage, and disease history (hypertension, dyslipidemia, and diabetes). We assessed the quality of propensity score matching using the Standardized Mean Difference (SMD), with a threshold of less than 0.1 considered acceptable.

## Results

### Baseline characteristics

Our study comprised 12,658 participants, among whom 821 individuals (6.48%) self-reported chronic kidney disease. The baseline characteristics were summarized in Supplemental Table 1. Depression data missing 9.0%, other data missing ranged from 0.3 to 4.1%. To address this, we applied data interpolation techniques. After PSM, we obtained 821 pairs of matched participants (Table 1). The mean age was 60.1 ± 9.0 years, 859 (52.3%) were male, 364 (22.2%) were fall within the last 2 years. Supplemental Fig. 1 and Supplemental Fig. 2 shows the ROC and SMD of fall accident rates in participant with/without CKD after PSM.

**Table 1:** Baseline characteristics of participants before/after propensity score matching

Characteristic	Participants before PSM			Participants after PSM		
	Non-CKD (*n* = 11,837)	CKD (*n* = 821)	SMD	Non-CKD (*n* = 821)	CKD (*n* = 821)	SMD
Male, n (%)	6236 (52.7)	391 (47.6)	0.101	429 (52.3)	430 (52.4)	0.002
Age, Mean ± SD	58.8 ± 9.5	60.2 ± 8.9	0.151	59.9 ± 9.1	60.2 ± 8.9	0.034
Pain status, n (%)	2835 (24)	344 (41.9)	0.389	332 (40.4)	344 (41.9)	0.03
Alcohol status, n (%)	4368 (36.9)	308 (37.5)	0.013	331 (40.3)	308 (37.5)	0.057
Night sleep duration, n (%)			0.177			0.008
≥ 6 h	8581 (72.5)	528 (64.3)		525 (63.9)	528 (64.3)	
< 6 h	3256 (27.5)	293 (35.7)		296 (36.1)	293 (35.7)	
ADL, n (%)			0.286			0.006
independent	10,368 (87.6)	630 (76.7)		632 (77)	630 (76.7)	
dependent	1469 (12.4)	191 (23.3)		189 (23)	191 (23.3)	
IADL, n (%)			0.258			0.034
independent	10,138 (85.6)	620 (75.5)		608 (74.1)	620 (75.5)	
dependent	1699 (14.4)	201 (24.5)		213 (25.9)	201 (24.5)	
Mobility, n (%)	252 (2.1)	32 (3.9)	0.104	31 (3.8)	32 (3.9)	0.006
Fall down experience, n (%)	1634 (13.8)	191 (23.3)	0.245	206 (25.1)	191 (23.3)	0.043
Depression, n (%)			0.236			0.02
<10	7746 (65.4)	443 (54)		435 (53)	443 (54)	
≥ 10	4091 (34.6)	378 (46)		386 (47)	378 (46)	
Cognition, n (%)			0.087			0.004
≥ 5.25	10,457 (88.3)	747 (91)		748 (91.1)	747 (91)	
<5.25	1380 (11.7)	74 (9)		73 (8.9)	74 (9)	
Toilet seat usage, n (%)			0.091			< 0.001
No	11,753 (99.3)	807 (98.3)		807 (98.3)	807 (98.3)	
Yes	84 (0.7)	14 (1.7)		14 (1.7)	14 (1.7)	
Hypertension, n (%)	2791 (23.6)	299 (36.4)	0.283	289 (35.2)	299 (36.4)	0.025
Dyslipidemia, n (%)	1386 (11.7)	164 (20)	0.228	167 (20.3)	164 (20)	0.009
diabetes, n (%)	753 (6.4)	87 (10.6)	0.152	91 (11.1)	87 (10.6)	0.016

*Note*: PSM, propensity score matching; CKD, chronic kidney disease; ADL, activity of daily living; IADL, instrumental activities of daily living

### Outcome analysis with PSM cohorts

**Fig. 2:**
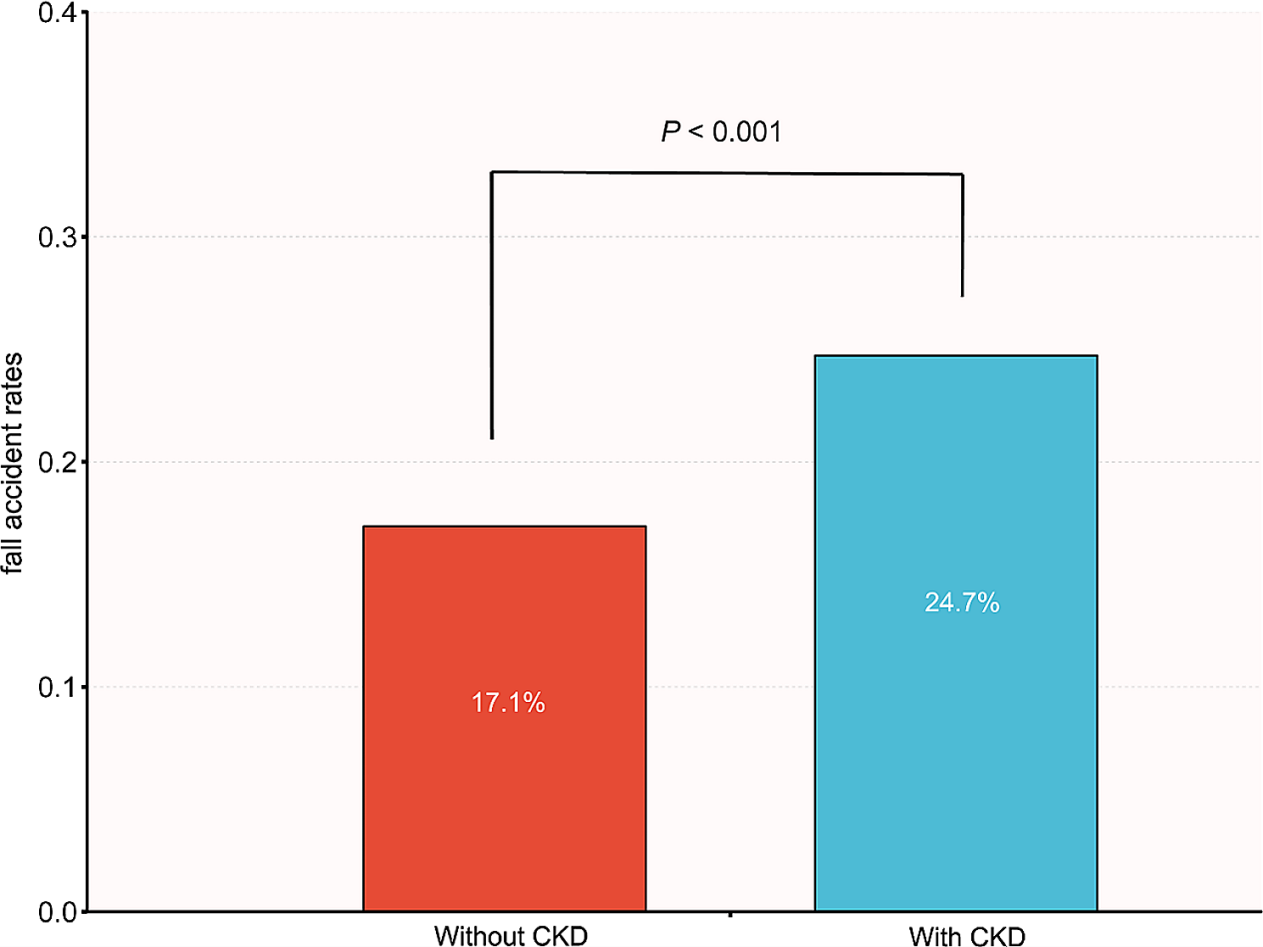
Bar graph of fall accident rates within participants without/with CKD. *Note*: chi-squared test: *P* < 0.001

Figure 2 showed that over the last 2 years, fall accident rates were 17.1% (2,028/11,837) among participants without CKD and 24.7% (203/821) among those with CKD. Notably, participants with CKD exhibited higher fall accident rates (chi-squared test: *P* < 0.001). Inverse probability weighting (IPW) analysis demonstrated a significantly higher rate of fall accidents in participants with CKD, with an OR of 1.28 (95% CI, 1.08–1.53, *P* = 0.005). The propensity score-matched fall accident rates for participants without CKD and participants with CKD were 19.6% and 24.7%, respectively. In the multivariable logistic regression analysis, the OR was 1.35 (95% CI, 1.07–1.7, *P* = 0.013) (Table 2).

**Table 2:** Associations between chronic kidney disease and fall accidents in the crude analysis, multivariable analysis, and propensity-score analyses

Analysis	Fall accidents	P - value
No. of events/no. of patients at risk (%)		
Without CKD	2028/11,837 (17.1)	
CKD	203/821 (24.7)	
Crude analysis - odds ratio (95% CI)	1.59 (1.35 ~ 1.87)	< 0.001
Multivariable analysis - odds ratio (95% CI) ^a^	1.26(1.06 ~ 1.51)	0.01
With inverse probability weighting ^b^	1.28 (1.08 ~ 1.53)	0.005
With matching ^c^	1.35 (1.07 ~ 1.7)	0.013
Adjusted for propensity scored ^d^	1.26 (1.06 ~ 1.5)	0.008

*Note*: CKD, chronic kidney disease

a Shown is the odds ratio from the multivariable logistic regression model, with adjusted for all covariates

b Shown is the primary analysis with a odds ratio from the multivariable logistic regression model with the same strata and covariates with inverse probability weighting according to the propensity score

c Shown is the odds ratio from a multivariable logistic regression model with the same strata and covariates with matching according to the propensity score. The analysis included 821 patients (821 who self-reported chronic kidney disease and 821 who did not)

d Shown is the odds ratio from a multivariable logistic regression model with the same strata and covariates, with additional adjustment for the propensity score

### Sensitivity analysis and subgroup analysis

**Fig. 3:**
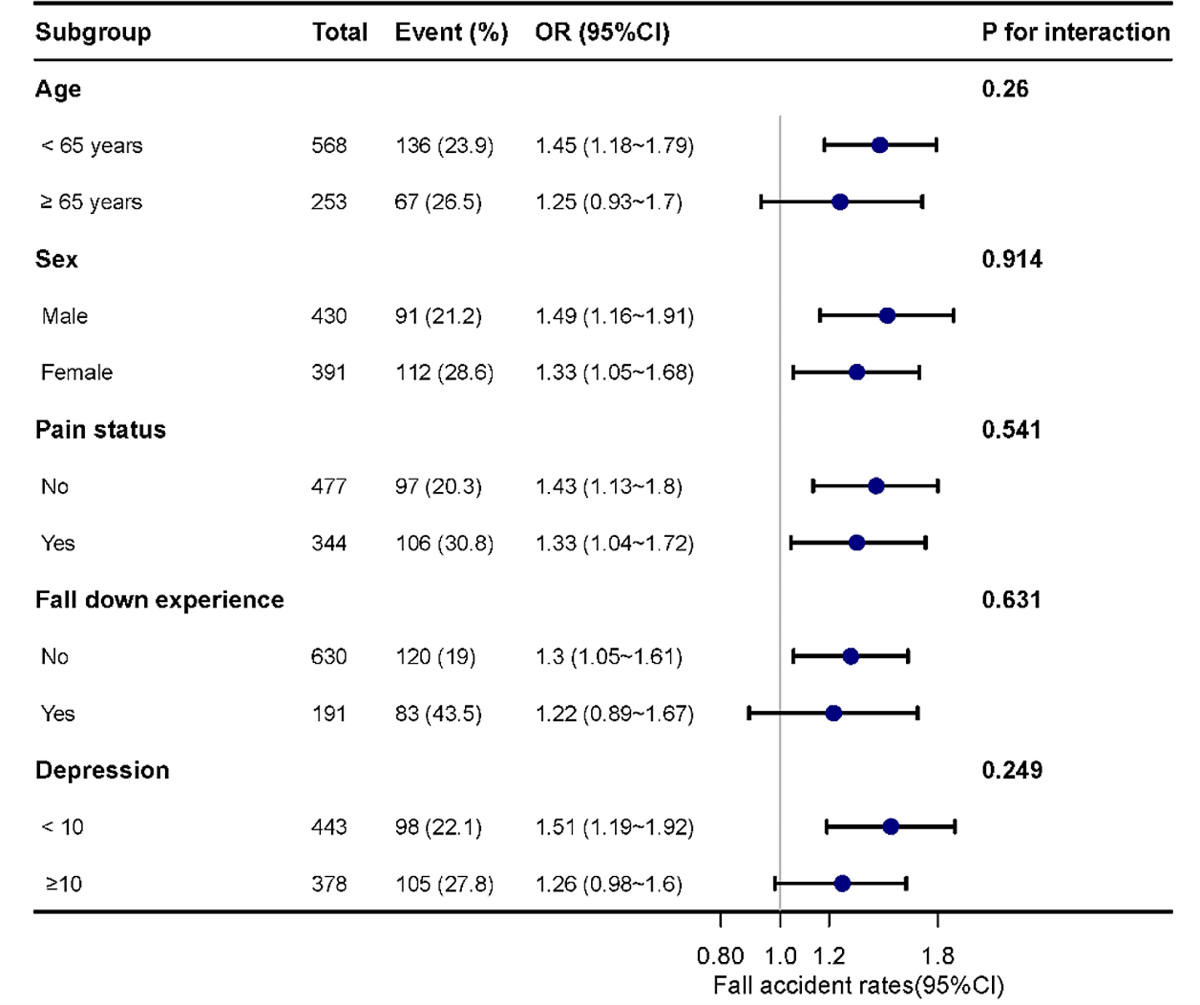
Forest plot of ORs of fall accidents in participants with CKD in subgroup analyses

We conducted a sensitivity analysis using four different models, as presented in Supplemental Table 2. This analysis aimed to further verify the stability of the results, and we found stability after adjustment using multiple models. As shown in Fig. 3, Subgroup analysis showed that the results remained robust and reliable.

## Discussion

In this retrospective propensity score-matched cohort study, participants with CKD exhibited a significantly higher risk-adjusted rate of fall accidents compared to non-CKD participants. This association remained consistent across sensitivity analysis and subgroup analysis.

To our knowledge, this is the first comprehensive examination of risk factors for falls within a substantial national sample of individuals with CKD in China. This is especially pertinent considering the growing prevalence of CKD in the country. Our findings reveal that individuals with CKD in our study tended to be older than the general population, and advanced age is a recognized pivotal risk factor for falls [11]. Falls among older individuals are of paramount concern due to their association with adverse outcomes such as fractures, diminished quality of life, loss of independence, and mortality. Despite the well-established focus on falls in the elderly, individuals with CKD often present with a constellation of comorbidities, potentially amplifying their vulnerability to falls. Therefore, our study includes middle-aged CKD patients to provide a comprehensive examination of the risk of falls within this population. In the subgroup analysis of our study, older adults with CKD did not exhibit statistically significant differences in fall rates compared to older adults without CKD. In contrast, middle-aged CKD patients showed a higher incidence of falls compared to their non-CKD counterparts. This difference may be attributed to muscle loss beginning at an earlier age among middle-aged CKD patients and age-related frailty among older non-CKD patients, which collectively contribute to a higher overall fall incidence. However, this conclusion requires further validation in larger sample sizes.

Our findings consistent with previous studies in the USA, which have reported a 20% higher risk of falls among CKD patients when compared to individuals without CKD [20, 21]. Other existing research has focused on end-stage kidney disease patients, particularly those undergoing hemodialysis, there is limited research on falls among early-stage kidney disease patients [14, 22]. However, it’s worth noting that some research has indicated that decreased renal function alone may not significantly elevate the risk of falls [23, 24]. Factors contributing to the heightened risk of falls in CKD patients may be attributed to physiological changes associated with CKD, rather than solely a decline in glomerular filtration rate.

There are numerous distinct causes for falls, including acute medical issues (e.g., postural hypotension, acute arrhythmias, transient ischemic attacks), activity or balance issues, household hazards (e.g., wet surfaces, poor lighting), and medication-related factors. Considering the cultural customs in China, people often use squat toilets rather than sitting toilets. The action of squatting and standing up can easily lead to postural hypotension, increasing the risk of falls. Additionally, flushing squat toilets can result in a wet bathroom floor, posing an environmental hazard and further elevating the risk of falls. Therefore, we hypothesize that not using sitting toilets may increase the risk of falls [9]. We have included toilet seat usage as a risk factor in our model to adjust for its potential impact.

In CKD patients, factors such as chronic inflammation, vitamin D deficiency, and proteinuria can contribute to muscle loss, compromising muscle strength and balance, thereby increasing the risk of falls [23, 25–28]. Furthermore, studies indicate that patients with chronic kidney disease often experience cognitive decline, which aligns with our study [29, 30]. Cognitive decline may contribute to impaired balance, thereby increasing the risk of fall accidents [31].

There are several limitations to this study. Firstly, CKD was defined based on self-reported physician diagnoses. Subsequently, we validated these diagnoses during the follow-up assessment conducted in the 2015 survey to ensure data accuracy. However, it is possible that some participants may still have undiagnosed CKD. Secondly, fall accidents were defined based on self-reported incidents of falling within the past two years, which could introduce the possibility of memory bias, as incidents without injury may be overlooked or forgotten. Thirdly, all participants in this study were of Chinese ethnicity. Therefore, there may be limitations in the generalizability of our study findings to other racial or ethnic groups. Fourthly, our study focuses on self-reported falls within a community setting, leading to an inability to precisely ascertain the specific causes of these fall incidents. Factors such as acute medical issues, activity-related or balance-related aspects, household hazards, and medication-related factors contributing to falls remain unexplored. While confounding elements are commonplace in retrospective research, they introduce a degree of bias into our findings. To mitigate these limitations, we implemented rigorous statistical methodologies, including PSM, Propensity Adjustment (PA), Overweighting (OW), and doubly robust estimation. Despite these efforts, the inherent retrospective nature of our study hinders complete bias elimination. To bolster the reliability and generalizability of our findings, future research should prioritize extensive prospective studies that comprehensively explore various factors influencing falls, thereby ensuring a more thorough understanding and validation of our conclusions. Fifthly, the study outcomes are limited to self-reported falls and do not encompass long-term consequences such as hip fractures/head injuries, pain, diminished quality of life, loss of independence, and mortality. Our primary focus is on the occurrence of falls as an incidental event. While we acknowledge that non-injurious falls are also worthy of attention, considering the cost-benefit ratio, prospective research is necessary to comprehensively explore these outcomes.

## Conclusion

Overall, our study demonstrates that population in China with CKD are at a significantly heightened risk of experiencing falls. This underscores the importance of giving heightened attention to the issue of falls within the CKD population, emphasizing the necessity for specific preventive measures and further research in this area.

d Shown is the odds ratio from a multivariable logistic regression model with the same strata and covariates, with additional adjustment for the propensity score.

### Electronic supplementary material

Below is the link to the electronic supplementary material.

Supplementary Material 1

## Data Availability

The datasets supporting the conclusions of this article are available publicly, http://charls.pku.edu.cn/pages/data/111/en.html.
