# Supplementary material for: Risk of fall in patients with chronic kidney disease: results from the China health and retirement longitudinal study (CHARLS)
Source: BMC Public Health. 2024 Feb 16;24:499. doi: 10.1186/s12889-024-17982-4 (PMC10873935; doi:10.1186/s12889-024-17982-4)
Supplement: Supplementary file 1 — Supplementary Material 1 [file 12889_2024_17982_MOESM1_ESM.docx]

Supplemental Table 1 Baseline characteristics of participants.

| **Variables** | **Non-CKD (n = 11837)** | **CKD (n = 821)** | **p** |
| --- | --- | --- | --- |
| Male, n (%) | 5601 (47.3) | 430 (52.4) | 0.005 |
| Age, Mean ± SD | 58.8 ± 9.5 | 60.2 ± 8.9 | < 0.001 |
| Pain status, n (%) | 2772 (24) | 337 (41.9) | < 0.001 |
| Alcohol status, n (%) | 4348 (36.9) | 308 (37.6) | 0.691 |
| Night sleep duration, n (%) | | | < 0.001 |
| ≥6h | 8295 (72.7) | 518 (64.7) |  |
| ＜6h | 3120 (27.3) | 283 (35.3) |  |
| ADL, n (%) |  |  | < 0.001 |
| independent | 9911 (87.2) | 627 (76.7) |  |
| dependent | 1453 (12.8) | 191 (23.3) |  |
| IADL, n (%) |  |  | < 0.001 |
| independent | 10089 (85.7) | 620 (75.5) |  |
| dependent | 1690 (14.3) | 201 (24.5) |  |
| Mobility, n (%) | 250 (2.1) | 32 (3.9) | < 0.001 |
| Fall down experience, n (%) | 1629 (13.8) | 191 (23.3) | < 0.001 |
| Depression, n (%) | |  | < 0.001 |
| ＜10 | 7086 (65.9) | 416 (54.3) |  |
| ≥10 | 3665 (34.1) | 350 (45.7) |  |
| Cognition, n (%) | |  | 0.057 |
| ≥5.25 | 10032 (88.5) | 724 (90.7) |  |
| ＜5.25 | 1302 (11.5) | 74 (9.3) |  |
| Toilet seat usage, n (%) | |  | 0.001 |
| No | 11708 (99.3) | 807 (98.3) |  |
| Yes | 83 (0.7) | 14 (1.7) |  |
| Hypertension, n (%) | 2791 (23.6) | 299 (36.4) | < 0.001 |
| Dyslipidemia, n (%) | 1386 (11.7) | 164 (20) | < 0.001 |
| diabetes, n (%) | 753 (6.4) | 87 (10.6) | < 0.001 |


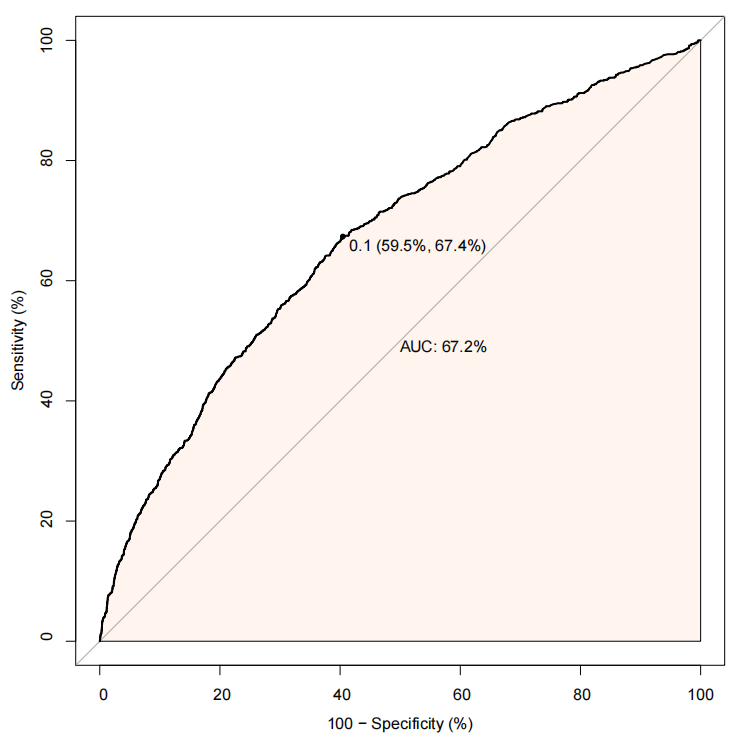


Supplemental Figure 1 ROC curve of fall accident rates in participant with/without CKD after PSM


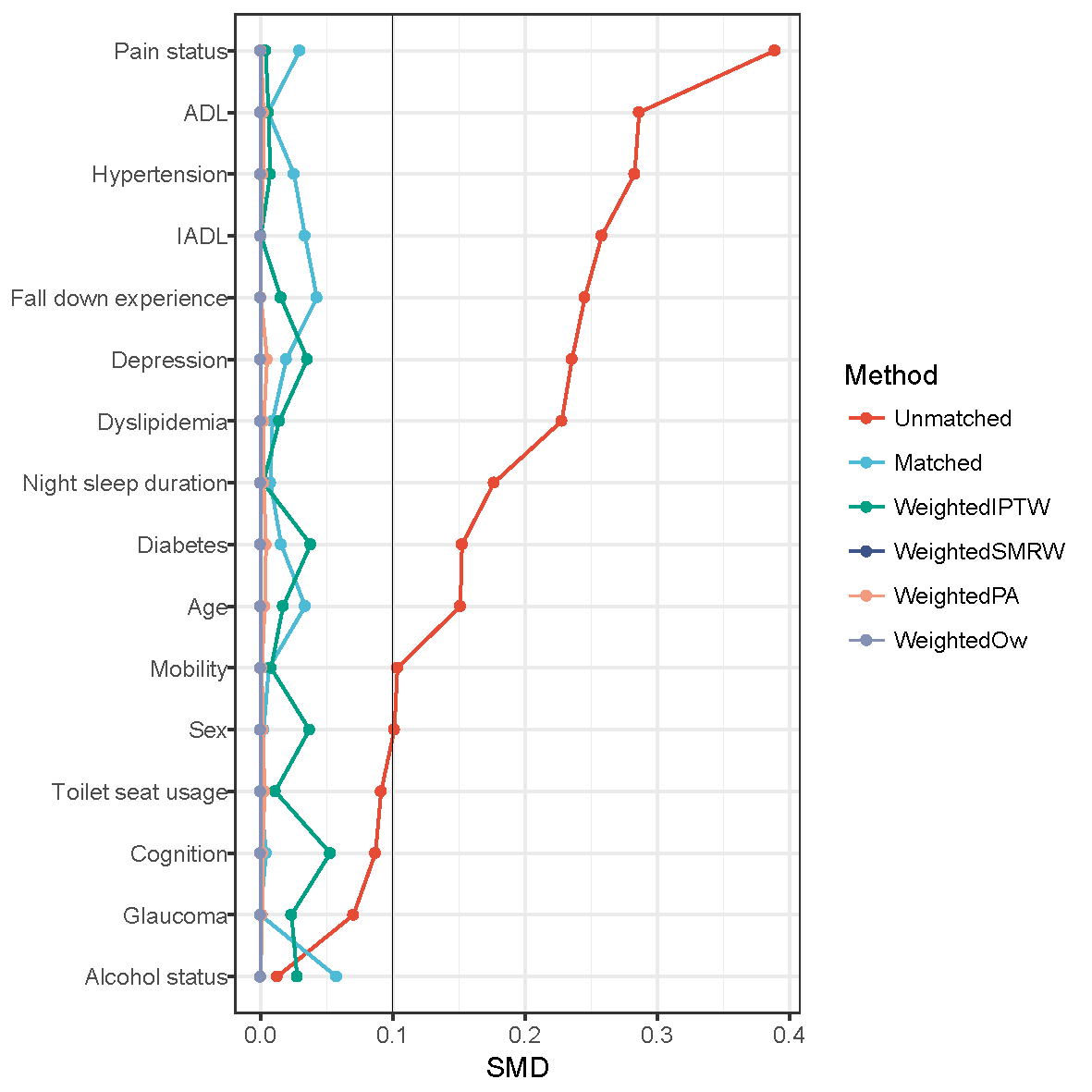


Supplemental Figure 2 SMD of fall accident rates in participant with/without CKD after PSM

Supplemental Table 2 Multivariable logistic regression analysis in participant with/without CKD

| **Variable** | **n** | **Model 1** | **Model 2** | **Model 3** | **Model 4** |
| --- | --- | --- | --- | --- | --- |
| Without CKD | 2028/11837 | 1(Ref) | 1(Ref) | 1(Ref) | 1(Ref) |
| With CKD | 203 /821 | 1.59 (1.35~1.87) | 1.59 (1.35~1.88) | 1.36 (1.15~1.61) | 1.36 (1.14~1.61) |

Model 1: No adjustment.

Model 2: Adjusted for sex, age.

Model 3: Adjust for the variables in model 2 plus alcohol status, night sleep duration, mobility, pain status, fall down experience, toilet seat usage, depression, cognition, ADL and IADL.

Model 4: Adjust for the variables in model 3 plus hypertension, dyslipidemia, and diabetes.
